# The Role of Soil Solarization in India: How an Unnoticed Practice Could Support Pest Control

**DOI:** 10.3389/fpls.2017.01515

**Published:** 2017-09-01

**Authors:** Harsimran K. Gill, Iqbal S. Aujla, Luigi De Bellis, Andrea Luvisi

**Affiliations:** ^1^Department of Entomology, Cornell University, Ithaca NY, United States; ^2^Department of Plant Pathology, Punjab Agricultural University Ludhiana, India; ^3^Department of Biological and Environmental Sciences and Technologies, University of Salento Lecce, Italy

**Keywords:** soil-borne pathogens, food safety, fungi, crop protection, plastic film mulching

## Abstract

Plant protection represents one of the strategies to fill the yield gap and to achieve food security, a key topic for India development. Analysis of climate risks for crops indicates that South Asia is one of the regions most exposed to the adverse impact on many plants that are relevant to inhabitants exposed to food safety risks. Furthermore, accumulation of pesticide residues in the aquatic and other ecosystems is becoming a significant threat in India. These perspectives require to develop programs of crop protection that can be feasible according to Indian rural development and pollution policy. Here we review the research works done on soil solarization in India. Soil solarization (also called plasticulture) is an eco-friendly soil disinfestations method for managing soil-borne plant pathogens. This is the process of trapping solar energy by moist soil covered with transparent polyethylene films and chemistry, biology and physical properties of soil are involved in pest control. So far, this technique is applied in more than 50 countries, mostly in hot and humid regions. India has 29 states and these states fall under five climatic zones, from humid to arid ones. We report pest management application in different climatic zones and their effects on production, weeds, nematodes, and pathogenic microorganisms. The analysis of soil temperatures and crop protection results indicate as environmental requirement for soil solarization fits in most of Indian rural areas. Soil solarization is compatible with future Indian scenarios and may support Indian national food security programs.

## The Need of Sustainable Crop Protection in India

The yield gap is a hiatus between actual production and the best yield achievable using genetic material and available technologies and management. It strongly depends on the possibility of farmers to access most appropriate genetic resources (seeds, plants), natural inputs (suitable soil and water), knowledge (practices and information), and technology (pest management, mechanization) (Godfray et al., 2010). This gap is not irrelevant and, in many areas worldwide, represents one of the main factors that prevent the achievement of food security (Pinstrup-Andersen, 2009). In parts of Southeast Asia, where water for agricultural purposes is usable, it has been estimated that average production of rice is just more than half of potential production using optimized input (Cassman, 1999).

Plant protection represents one of the strategies to fill the yield gap. Control of pests and microbial pathogens, at least applying agronomic methods, is de-facto mandatory, and the role of chemicals in agriculture in the last century is crucial. Worldwide, the pesticide consumption is about two million tons per year. India, with its average pesticide usage rate of 0.5 kg per hectare, contributes 4% to the world’s total pesticide consumption. More than 80% of the agrochemicals used in India are classified as insecticides, while 15% are herbicides and 2% are fungicides (Agarwal et al., 2015). Though the pesticide consumption rates are high in the most productive areas of India and comparable to the high amounts used per hectare in the other parts of the world (Europe or United States), vast territories of poor crop production areas with nil or very low pesticide consumption pulls down the national average. With the emphasis on food security, these territories are the focus of the Indian government for realizing the objectives of National Food Security Act. Even in the high productivity areas, higher yielding varieties and better management practices are being deployed to further improve the marginal gains in rice, wheat, maize, pulses, and other crops. India has started reaping benefits of these efforts (Ray et al., 2013). Pesticides have played a significant role in realizing these benefits. But this increased pesticide usage has led to the accumulation of pesticide residues in the aquatic and other ecosystems. Contamination of drinking water sources with these pesticide residues is becoming a significant threat in India (Pillai and Rao, 1974; Khan et al., 1997, 2000; Gill and Garg, 2014; Agarwal et al., 2015). Moreover, beside economic costs for their application (not irrelevant for many populations, such those of developing countries), the environmental impact of agrochemicals are assuming a central role in public debate worldwide and the search of environmental friendly protection techniques is primary (Gill and Garg, 2014).

Among plant protection techniques, soil solarization (also called plasticulture) may play a significant role in the next years because it could be considered a sort of paradigm of sustainable crop protection method, particularly useful for countries such as India. The low environmental impact (mainly due to plastic film disposal, avoidable if biodegradable films are used), the wide-spectrum of actions against pathogen and the environmental requirement for effective solarization treatments seem fit perfectly with the Indian needs of plant protection. Moreover, the low costs of investment and application (mainly limited to soil mulching machine), the no-need of high-tech infrastructures or farm ability, the yield increase and perspective in climate changes could strongly support underdeveloped productive areas of India and sustain their food security.

## Soil Solarization: A Brief Compendium

First reports of soil solarization practice date back to late ‘800 in German Empire and United States, where it started being used commercially. Solarization is a chemical-free way of controlling pests such as pathogenic microorganisms (mainly fungi, bacteria, and nematodes), insects, and wild plants in the soil before crops planting (Katan, 1987; McGovern and McSorley, 1997; Gill et al., 2009). Solarization technology does not release any dangerous chemical residue in the ground; it is a safe, simple, effective, and eco-friendly tool for the home gardens and fields (Kapoor, 2013). This pre-plant method involves soil heating by capturing solar radiations for 4–6 weeks during the summer time when the soil receives the maximum sunlight. Soil intercept the energy radiated from the sun and its temperature rise to the level that is deadly to many soil-borne pathogens. It improves soil texture and the nutrients availability in the soil which are essential for plants growth and development. Unfortunately, in India, most of the farmer and rural people are not aware of the applications of soil solarization technology. There are a need of training workshops and mass awareness programmers for popularizing this technology among the farmers and rural communities (Kapoor, 2013). The primary commercial use of solarization involved sunny and warm lands. The mechanism of action is based on the increased soil temperature (commonly in the range of 45–55°C) at soil depth of 5-cm underneath transparent polyethylene sheets causes plant pathogens mortality (Katan, 1981). Besides the reduction in soil-borne pathogens, soil solarization also leads to increased growth response (IGR) of plants (Katan, 1981; Horiuchi, 1984). Soil solarization also integrates well with other techniques such as mulching, and soil amendments to enhance its overall effectiveness against pests and, increase crop yield (D’Addabbo et al., 2010).

Diversity of soil, climate, and social and economic values of the human population leads to the variety of the cultivated crops in India. Rice, wheat, barley, maize, cotton, groundnut, sugarcane, pulses, tobacco, pigeon pea, soybean, sorghum, cherry, strawberry, apple, and tomatoes are most commonly cultivated crops in India. However, different crops are grown in different states of India depending on the soil type, climate conditions, temperature, and rainfall, etc. More information on the main crops grown in India can be accessed at these web links^1^^,^^2^. Few commonly associated weeds in these cultivated crops include *Phalaris minor, Cyperus rotundus, Cynodon dactylon, Acrachne racemosa, Avena* spp., and *Orobanche* spp. Most common soil-borne fungal and nematode pathogens include *Sclerotinia* spp., *Rhizoctonia* spp., *Phytophthora* spp., *Macrophomina* spp., *Fusarium solani, Fusarium oxysporum, Pythium* spp., *Sclerotium* spp., *Meloidogyne incognita, Heterodera* spp., *Pratylenchus* spp., and *Rotylenchus* spp.

### Weeds Control

The literature points to the success of solarization in managing weeds even up to 98% in corn (Ahmad et al., 1996), while on the other hand 90% crop damage due to weeds alone is also reported in unsolarized control plots (Elmore et al., 1997). Worldwide, solarization has managed to control annual weeds such as annual bluegrass, *Ageratum* spp., *Amaranthus* spp., barnyard grass, cogongrass, common purslane, *Digitaria* spp., *Portulaca* spp., redroot pigweed, *Setaria* spp., and many others (Daelemans, 1989; Benlloglu et al., 2005). The weeds elimination also prevents the growth and widespread of pathogenic microorganisms or insects which may spend their lifecycle on wild plants (Stapleton and DeVay, 1986). Generally, winter wild plants are easier to eliminate, whereas summer wild plants such as *Cyperus* spp. or *Convolvulus arvensis* showed a good resistance against the disinfection treatment (Katan, 1981).

### Microbial Pathogen Control

Damping-off, root rot, and wilt are economically damaging plant diseases. Soil solarization has managed to manage the causal organisms (fungus or bacteria) of these diseases. Few successfully managed soil-borne fungal and bacterial in different cropping systems include *Rhizoctonia* spp., *Fusarium* spp., *Sclerotinia* spp., *Macrophomina* spp., *Phytophthora* spp., *Verticillium* spp., *Agrobacterium tumefaciens, Clavibacter michiganensis, Pythium* spp., and *Streptomyces scabies* (Ahmad et al., 1996; McGovern and McSorley, 1997; Chellemi and Mirusso, 2006; Gelsomino et al., 2006).

### Nematodes and Insect Control

Solarization can manage broad range of nematodes (roundworm or threadworm) like spiral nematode (*Helicotylenchus digonicus*), root-knot nematode, reniform nematode (*Rotylenchulus reniformis*), ring nematodes (*Mesocriconema* spp.), pin nematode (*Paratylenchus hamatus*), lesion nematode (*Pratylenchus* spp.), cyst nematode (*Heterodera glycines* Ichinohe), sting nematode (*Belonolaimus* spp.), dagger nematodes (*Xiphinema* spp.), stubby root nematode (*Paratrichodorus minor*), stem and bulb nematode (*Ditylenchus dipsaci* Kuhn.), potato cyst nematode (*Globodera rostochiensis*), northern root-knot nematode (*Meloidogyne hapla*), and sugar beet cyst nematode (*Heterodera schachtii*) (Abdel-Rahim et al., 1988; Stapleton and Heald, 1991; Elmore et al., 1997; McGovern and McSorley, 1997). Insects (mainly stored grain pests) have been controlled by soil solarization to some extent as well. Soil solarization was observed to change soil chemistry which may weaken or kill some kinds of soil organisms (McFarlane, 1989; Elmore et al., 1997; Lale and Ajayi, 2001; Minarro and Dapena, 2003; Gill and McSorley, 2010; Summers et al., 2010).

### Effects on Soil Chemistry and Microbial Ecosystem

Generally, the highest temperature (more than 50°C) can be achieved during daytime only in the upper 5 cm of soil. At lower depth (10–15 cm) soil temperature may pass 40°C in warm environment and during summer, and more than 35°C can be achieved up to 30 cm depth (Stapleton, 1997). However, the effect of energy emitted by sun on soil temperature is strongly dependent to climate and weather.

Plasticulture enhanced the availability of useful constituents (potassium, nitrogen, magnesium, and manganese) in easier to assimilate compounds that can increase pathogen tolerance, and eventually increase the yield of crops by altering chemistry of soil and its physical characteristics through increased breakdown of organic material (Ahmad et al., 1996; Elmore et al., 1997; Chellemi and Mirusso, 2006). Nitrogen transformations (nitrification and ammonification) were found to be greater in treated fields compared with non-solarized ones (Stevens et al., 1991), while integration of manure with soil solarization found to be greatly increased the C, N, and P pools. This integration could be used as key plan to defend farming lands from soil mistreatment and to enhance pest control while maintaining soil fertility (Gelsomino et al., 2006). Plasticulture helped at increasing the concentration of NO_3_^-^ and NH_4_^+^ up to six times compared to non-solarized soils. It also increased the P, Ca^+2^, Mg^+2^, and electrical conductivity, which was linked to the greater quantity of ions released following decomposition and mineralization of organic matter (Chen and Katan, 1980) and increased yield of many crops as observed in solarized plots (Stapleton et al., 1985).

Most of the soil microorganisms are beneficial and enzymatic activities are considered as biosensors of soil health (Aon and Colaneri, 2001; Aon et al., 2001; Poll et al., 2006; Velmourougane et al., 2013) thus impact of soil treatment on microbial ecosystem is crucial. Effectiveness of soil solarization was also related to effect of treatment on availability of chemical compounds originated from degradation of mesophilic microorganisms. This increment of nutrients can lead to an increased growth of benefic antagonists of plant pathogens that were commonly reported in treated soils (Stapleton and DeVay, 1995; Weller et al., 2002). Desirable effects are also associated with shifts in soil microflora caused by reduced pressure on soil microbiota by microorganism-grazers which are stressed by plasticulture (Gupta and Yeates, 1997). Generally, heating treatment caused significant changes in biodiversity of soil microflora (D’Addabbo et al., 2010), while studies on the fungi and bacteria communities of plasticulture-treated soils showed lower cumulative carbon metabolic activities than untreated ones (Luvisi et al., 2015). Thus, plasticulture causes shifts in microflora composition which can consume carbon substrates at lower efficiency as control. The measurement of carbon source utilization richness (which reflect different microorganism metabolism) and the Shannon’s index of carbon source utilization (related to functional diversity) were also slightly affected by solarization (Luvisi et al., 2015). Furthermore, soil composition may also interfere with soil biota during solarization due to decomposition of organic material that can produce fizzy chemicals poisonous to microorganisms in soil (Gamliel et al., 2000). Contrasting reports (positive or negative shifts) in soil bacteria were reported, with *Bacillus* species as predominant surviving after soil solarization treatments (D’Addabbo et al., 2010), while reduction of soil rhizobia was generally followed by quick recover (Linke et al., 1991).

The integration of soil biofumigation with plasticulture (biosolarization) is considered a sustainable option for crop protection (Mauromicale et al., 2010; Domínguez et al., 2014), and biofumigation may also increase growth of beneficial microorganism (Camprubí et al., 2007). Further study conducted in southern Italy, documented that integration of soil solarization with organic amendments keep microbiota and soil enzymatic properties protected from deleterious heating effects (Scopa and Dumontet, 2007).

## Applications of Soil Solarization in India

An incredible amount of work is being done on solarization throughout the world with humid and hot climate. Compared to this, in India, less work is being done so far, and if it is done, it did not get in the limelight. Most of the work done on soil solarization in India did not get published online. Our literature survey included articles, books, and conference papers (retrieved from Scopus database, Research gate, and Google Scholar). We also carried out interviews with Indian scientists and technicians who worked on soil solarization in India. Interviews were mainly done by email survey, collecting stored data, and personal communications about local applications of soil solarization. In the following section of this review, the research work done in various states of India is discussed. These states fall under the humid, moist sub-humid, dry sub-humid, semi-arid, and arid climatic zones of India (**Table 1**) based on administrative districts as given by Raju et al. (2013). Air and soil solarization temperatures attained at different sites in these states are shown in **Table 2**.

**Table 1:** Classification of climate zones of India and locations (state and city) in which soil solarization trials were carried out.

Climate zone	State (city)
Humid	Karnataka (Shimoga)
	Manipur (Manipur)
Moist sub-humid	Himachal Pradesh (Shimla, Solan)
	Kerala (Trivandrum)
	Uttarakhand (Haldwani, Pantnagar)
	West Bengal (Burdwan)
Dry sub-humid	Madhya Pradesh (Akola, Gwalior, Jabalpur)
Semi-arid	Delhi (Delhi)
	Karnataka (Bangalore)
	Gujarat (Gandhinagar)
	Punjab (Abohar, Jalandhar, Ludhiana)
	Tamil Nadu (Chidambaram)
	Telangana (Hyderabad)
	Uttar Pradesh (Allahabad, Lucknow)
Arid	Rajasthan (Jodhpur)

**Table 2:** Soil solarization temperatures achieved in different climate zones of India.

State	Soil^∗^	PE (μm)^∗∗^	Solarized soil		Control soils		Reference
			Max^∗∗∗^	Min	Max	Min	
**Moist sub-humid**							
Himachal Pradesh	CL	25	49	25	37	21	Gupta and Khosla, 2007
Himachal Pradesh	LS	25	47	18	37	15	Sharma et al., 2005
Uttarakhand	–	25	55	–	44	–	Singh, 2008
Kerala	–	50	42	–	36	–	Nair et al., 1990
West Bengal	CL	25	48	39	39	30	Chakraborty et al., 2009
**Dry sub-humid**							
Madhya Pradesh	LS	110	52	–	46	–	Verma et al., 2010
**Semi-arid**							
Delhi	LS	100	48	34	40	32	Kumar et al., 1993
Uttar Pradesh	–	47	54	–	45	–	Dwivedi, 1998
Telangana	V	100	48	38	39	33	Chauhan et al., 1988
Tamil Nadu	CL	200	46	–	28	–	Jayaraj and Radhakrishnan, 2008
**Arid**							
Rajasthan	LS	50	51	45	53	49	Lodha et al., 1997

*Temperature attained at a site representative of a climatic zone mentioned in this paper. ^∗^Type of soil: CL, clay loam; LS, loamy sand; V, vertisol. ^∗∗^Polythene sheet thickness. ^∗∗∗^Temperature (°C) at 10 cm depth.*

### Humid Climate Zone

Manipur and parts of Karnataka states fall into this climate zone. The main plants cultivated in this zone are rice, maize, jowar (*Sorghum* spp.), ragi, pulses [tur (*Cajanus cajan*), and gram], and other oilseed crops. In Manipur, soil solarization was more efficient in managing pigeon pea wilt, caused by *Fusarium udum* Butler compared to organic amendments (FYM or poultry manure) or intercropping with maize (Devi and Chhetry, 2013). In Southern peninsular India, Karegowda et al. (2008) found soil solarization alone is not giving a better cost–benefit ratio in reducing the incidence of damping-off disease (*Pythium aphanidermatum*) of FCV tobacco. The economic returns improve when solarization combines with twice drenching of soil with cow urine and a spray of Ridomil MZ 72 (0.2%). Excessive rains in this climate zone hamper the exclusive use of solarization, but it can be beneficial when used in conjunction with other management practices. Not many known studies were conducted in humid zone, but soil solarization could be used as a potential pest management option for managing pests of chili, cardamom, tea, coconut, and coffee.

### Moist Sub-humid Zone

Uttarakhand, Himachal Pradesh, West Bengal, and Kerala states come under moist sub-humid climatic zone.

#### Uttarakhand

It is mainly a hilly state located in the northwestern part of India. Cultivable land constitutes only 20% area of the state. Rice, coarse cereals, wheat, groundnuts, soybean, pulses, and oilseeds are common crops cultivated in Uttarakhand. Few solarization studies were carried out in Nainital and Pantnagar districts (as given below) on soybean and sorghum crops and beneficial results were found.

• Haldwani (Nainital district). With solarized soil attaining temperatures of 41°C at 6 cm soil depth, Dubey et al. (2009) reported a decline in the incidence of charcoal rot of soybean due to *Macrophomina phaseolina* infection from 85% in the unsolarized soil to 70% in solarized soil. Combining soil solarization with neem powder cake further caused reduction of rot disease to 60% with the additional benefits of increased seedling growth and biomass.
• Pantnagar. Solarization from mid-May to mid-June with 25 μm transparent polyethylene sheets rises of 10°C the average maximum temperature of the soil at 5 cm soil depth over the non-solarized soil. The temperatures higher than 45 and 50°C were measured at 5 and 10 cm depths for the most duration of soil solarization (**Table 2**). Plasticulture alone or integration with *Trichoderma harzianum* (TH 43 and TH 39) and *Pseudomonas fluorescens* (PSF 27) reduced disease severity of anthracnose of sorghum, increased plant height and collar diameter (Singh, 2008).

#### Himachal Pradesh

Himachal Pradesh is also a hilly state and lies on the western side of Uttarakhand. Its climate is also similar to Uttarakhand with a same temperature range in the months of May to June. Most cultivated plants grown in Himachal are wheat, barley, maize, rice, with apple as the main cash crop. Solarization studies in Himachal mainly focused on fruit crops and found effective to manage many kinds of plant diseases.

• Solan. Solan have moderate climate during summer with maximum daily temperatures 21–37°C with little cloud cover. Gupta and Khosla (2007) observed that soil temperature increased by 13–14, 4–5, and 2–3°C at soil depth of 8, 15, and 30 cm, respectively, when irrigated clay loam soil was covered with 25 μm thick transparent plastic film for 3 months from April to June as compared to temperature achieved in non-solarized soil. The average temperatures increase of 19–57% at 15–8 cm soil depths in solarized plots of cherry. It reduced the crown gall (*A. tumefaciens*) incidence in cherry rootstock colts from 45% in non-solarized to 15% in solarized plots. The effect of solarization in non-irrigated plots was less effective (27%). Further integration of solarization with *Bacillus subtilis* BCA-6 reduced crown gall incidence to 11%. Raj and Sharma (2005) found that 40 days of plasticulture with transparent plastic film (25 μm thickness) was effective for controlling collar and root rot of strawberry which was caused by *Sclerotium rolfsii* during June to July. The treatment caused absolute death of sclerotia of the pathogen at 7 cm soil depth, but the effectiveness of plasticulture was lower when soil depth increase; plasticulture for shorter time (40 days) caused only 80 and 73% mortality of sclerotia of *S. rolfsii* at 15 cm depth. The effectiveness of solarization was enhanced with root dipping of strawberry runners in association of chemical treatments with carbendazim 12% and mancozeb 63% (0.2%) or thiophanate methyl (0.1%); in this case, cultivation in treated soils resulting in no infected plant. Raj and Sharma (2009) found inoculation of apple with AMUHF1 isolate of vesicular-arbuscular mycorrhiza and AZUHF1 isolate of *Azotobacter chroococcum*, and followed by cultivation in treated soil with clear polyethylene mulch of 25 μm thickness for 40 days in summer months to be effective. White root rot (*Dematophora necatrix*) was totally controlled in treated plots compared to 34–35% of incidence in untreated ones. Root and shoot length of the apple saplings was higher in solarized plots compared with sterilized plots. In mid-hill rainfed weather conditions of Solan, Sharma et al. (2011a) reported solarization of clay loam soil with 100 μm thick, UV-stabilized, transparent polyethylene sheets rise the maximum temperature to more than 48°C while the minimum temperature exceed 29°C up to 25 cm depth in warmer months. With black polyethylene mulch, the maximum average soil temperature was 40°C in treated soil. They found that combining rhizosphere soil inoculation with arbuscular mycorrhizal (AM) fungi and plasticulture using black plastic mulching was the efficient method for citrus nursery protection under mid-hill rainfed weather conditions of the State. The AM fungi especially *Glomus fasciculatum* (*Thaxter sensu* Gerdemann) and *A. chroococcum* (especially AZ1 strain) significantly increased plant growth, caused beneficial shifts in microflora composition of the rhizosphere and increase content of essential elements such as nitrogen, phosphorous, potassium, and zinc in leaf compared with other soil treatments. Sharma et al. (2011b) also observed similar technique to be beneficial for apple nursery too.
• Shimla. Sharma et al. (2005) found that long-duration plasticulture treatments during May to June were effective for management of soil-borne pathogens in temperate areas. In their study, soil solarization was conducted for 2, 4, 6, 8, 10, and 12 weeks period in April to June, using polyethylene sheets of 25 μm thickness. Plasticulture was most effective during May but satisfactory control of pathogens was also obtained in June, while treatments in April lead to worse results. Twelve weeks of plasticulture in fruit nurseries caused the highest decrease in population of most soil-borne microorganisms, while survival up to 40% of propagules of *D. necatrix*, the agent of white root rot disease, was observed at 15 cm depth.

#### West Bengal

West Bengal lies in the eastern part of the country with the Bay of Bengal lining its southern border. Rice, wheat, jute, potato, and sugarcane are the most common grown crops in this state. This state is big supplier of potato (90%) and jute (66%) for India. The other crops include vegetables, maize, barley, oil seeds, and pulses. Maximum and minimum air temperature of 33 and 27°C was reported in West Bengal during the months of May to June. Major solarization work is done on vegetables and jute with promising results.

In Burdwan, Chakraborty et al. (2009) found the provision of additional 50 μm polyethylene sheet for 2 h at peak hot time of the mid-May to mid-June over and above the transparent polyethylene sheet (25 μm thickness) further raised the soil temperature from 39 to 48°C in solarized clay loam type soil (**Table 2**). In untreated soil, it can change from 30 to 39°C up to 10 cm depth. Soil covered with polyethylene sheet markedly got temperatures of 46°C (average), while the non-solarized treatments differed almost 9°C lower. They observed 71 and 83% reduction in fusarial wilt (*F. solani*) of eggplant in solarized plots alone, in two sequential growing seasons. Further combining soil solarization with *T. harzianum* or *Trichoderma viride*, neem and bavistin provided 100% inhibition of fusarial wilt of eggplant.

Transparent and black polyethylene sheets of 200–400 gauge thickness raised the soil temperature up to 54°C in 0–5 cm soil depth compared to 38–40°C in control bare plots. Sprouted grass, broad-leaved weeds, and sedges were killed in jute crop, and weed emergence was reduced by 45–78%. Soil solarization can be practiced by growers provided that the cost of polyethylene sheets is low, and its safe disposal is easy (Ghorai, 2007).

#### Kerala

This Indian state famous as “Spice Garden of India” lines the western side of South India. Agricultural sector is mainly focused on coconut, tea, coffee, cashew, and spices. Few solarization studies were conducted and more work can be done in future to sustain the ecology of this state. In trial test at Trivandrum, root nodulation increased by 105%, infection caused by mycorrhizal fungi rise by 20% and yield was also increased by 24% compared to unsolarized soil (Nair et al., 1990). Transparent LDPE (low-density polyethylene sheets) of 50 gauge thicknesses was used as soil cover for 30 days. It reduced the weed growth and increased rice yield in tests carried out in Bhubaneswar (Orissa). Higher yield was documented in transparent sheets of 200–400 μm thickness for 1 month, followed by soil coverage with black sheets. The period of soil cover with plastic sheets should be decided based on soil and air temperatures, and solar radiations to kill soil pests and weeds (Khan et al., 2003).

### Dry Sub-humid Zone

#### Madhya Pradesh

This state is sited in the central part of India. The weather of Madhya Pradesh is classified as subtropical; springs are generally hot and dry, while consecutive monsoon rains characterized the summers. Winters are cool and relatively dry, with rainfall of about 54 inches. More than 30% area of this state is under forests. Cereals, pulses, and oilseeds are widely cultivated. In few districts of this state, farming of cotton and sugarcane is carried out. Loamy sand soil is the most common soil type reported in this state.

• Jabalpur. Jabalpur is located at an elevation of 415 masl with average air temperature range between 41 and 21°C during April to June. Solar heating of nursery seedbeds with the top soil consisting of a mixture of loam, sand, and farm yard manure was done for 1 month, with transparent sheet of 0.11 mm of thickness. Maximum temperatures achieved at 5 cm depth exceed 60°C with a mean difference of 10°C above control. The highest difference of temperature was 9°C whereas the minimum was 4°C with average difference of 6°C at 10 cm depth (**Table 2**). This enabled complete elimination of the soil inoculum of *F. solani, F. oxysporum, Penicillium*, and *Rhizopus* from upper 0–10 cm depth. The population of *Aspergillus, Trichoderma*, nematodes, AM fungi and bacteria decreased more in 0–5 cm soil depth and to a lesser extent in 5–10 cm depth. Solar heating also resulted in increased vegetative growth of seedlings of *Gmelina arborea* and *Tectona grandis* compared to the non-solarized control (Verma et al., 2010).
• Akola. Field studies were conducted on Citrus at Akola, at 5 and 15 cm soil depths. It was documented a temperature gain of 9–11°C at 5 cm depth, causing a control of *Phytophthora* spp. inoculum from 38.2 to 2.0 cfu (Gade and Giri, 2005).
• Gwalior. Field experiment was conducted with various agronomical and physical weeds control methods, plasticulture (for 45 days), green manuring, and mulching by green biomass. The worse control of wild plants was obtained in non-solarized plots, followed by practices such as green manuring, stale seed bed, mulching, and deep plowing. Soils treated with plasticulture achieved the better results, resulting as one of the best chemical free weed management method (Arora and Tomar, 2012).

### Semi-arid Zone

#### Uttar Pradesh

This state is in north-central region of India. The average maximum and minimum air temperature in the month of May to June are 40 and 25°C, respectively. Cereals, oilseeds, pulses, and potatoes are the most cultivated plants and sugarcane is the main cash crop throughout the state.

• Allahabad. Soil solarization with 40 μm thick transparent polyethylene sheets during May to June 2012 at Allahabad increased average maximum soil temperature than unmulched one by more than 15°C up to 10 cm depths (Kamaluddeen and Simon, 2013).
• Lucknow. Plasticulture performed for 21 days in June raised the temperature of solarized plot by 8, 7, and 14% compared to unsolarized plot at 5, 10, and 15 cm, respectively. Plasticulture caused significant detrimental effects in the population of *F. oxysporum* f.sp. *ciceris* and *F. udum*, the agents of wilt in chickpea and pigeon pea, respectively. After 21 days of solarization, the population of both pathogens was reduced by more than 72% at 5 cm, by >60% at 10 cm and by >53% at 15 cm soil depths. The population of *M. incognita* was also reduced significantly by 58% (Shukla and Dwivedi, 2011). In another solarization study, the upper soil layer (0–8 cm) reached 54°C, while no more than 45°C were recorded in untreated soil (**Table 2**). The inoculum of *Colletotrichum falcatum* and *Fusarium moniliforme*, two widespread pathogens of sugarcane, was almost eliminated up to 24 cm soil depth. Dwivedi (1998) also reported complete elimination of propagules of *Rhizoctonia solani* and *S. rolfsii* at 24 cm of soil depth. Dwivedi (1991b), Dwivedi R. (1993), Dwivedi S.K. (1993) reported that soil solarization (depth of 16 cm) reduced the inoculum of many species of *Fusarium* and *R. solani* related to wilt diseases of guava. Similar results are also reported the inhibition and eradication of charcoal rot pathogen (Dwivedi and Dubey, 1987; Dwivedi, 1991a) and tomato wilt pathogen (Dwivedi, 1991b).

#### Punjab

It is a northwestern state, and a major food bowl of India. Wheat and rice are the main cereal crops followed by cotton and citrus. Potato, sugarcane, maize, pulses, and vegetables are also cultivated. Temperature in summers exceeds 40°C with low of 26°C. Solarization has been tried on potato and high-valued vegetable crops in protected cultivation.

• Abohar. A field experiment was conducted in Abohar, Punjab and it was reported that transparent plastic sheets for 1 month showed good control of pathogens, and it drastically increased (more than 90%) the soybean yield (Singh, 2006).
• Jalandhar. Soil solarization reduced major annual weed *Coronopus didymus* and total number of weeds, but had no effect on the population of *C. rotundus* on true potato seeds. Increased yield showed that soil solarization involved elevation of plantlets in treated nursery beds and their movement to the untreated plots is advantageous for growing potato plants (Kumar and Sharma, 2005). Soil beds for potato growth were covered with 50 μm LDPE sheets for 4 weeks in June at Jalandhar. Plastic films rise soil temperature up to 53°C at ground level, 51, 44, and 39°C at 5, 10, and 15 cm depth, increasing soil temperature by 11, 9, 6, and 4°C at respective soil depths in unsolarized control plots. This led to increased yield from micro and mini-tuber potato crop (Singh et al., 2009). A similar trial was carried out to estimate the quality and quantity of potato seed. The weed populations were reduced by 95% and its fresh weight by 97%. Nutrients availability was increased by 18, 63, and 27% at planting and 16, 22, and 15% at harvesting, respectively. Plasticulture also increase the yield of micro-tuber (32% of increase) and mini-tuber (15% of increase) compared to untreated soil (Singh et al., 2012).
• Ludhiana. Solanaceous and cucurbitaceous vegetable plants are cultivated in net houses in the Punjab state of India. The severe attack of soil-borne pathogens such as sclerotinia wilt (*Sclerotinia sclerotiorum*) and root-knot nematode was detected during a disease survey of solanaceous and cucurbitaceous vegetable crops in greenhouses in many locations in the Punjab state of India during the cold seasons (Aujla et al., 2011). Application of 50 μm LDPE sheets both on the soil surface and roof top of the net house enabled the average minimum temperature of wet, sandy loam soil to remain above 41°C from 5 to 40 cm depth for more than 288 h in the last fortnight of 30-day solarization period in the months of May to June (Iqbal Aujla, unpublished data).

#### New Delhi

New Delhi is a capital state of India and it has a characteristic semi-arid to arid climate with average temperature range of 26–39°C in May to June. Soil type is sandy loam. Wheat, paddy, jawar, and bajra are the main crops grown in Delhi, along with vegetables includes cauliflower, cabbage, carrot, spinach, mustard (leaves), okra, and tomato. It has been hub of solarization studies in India since 1990s for control of soil-borne pathogens and wild plants, but the technology spread is not widespread. Being the administrative center of Indian agriculture, it needs to put more concerted efforts in spread of solarization technology in India.

At New Delhi, mulching of sandy loam soil for 3 weeks with transparent polyethylene sheets of 60 μm thickness increased the temperature by 16%. Solarization (when temperature exceeded 40°C) reduced the population densities of root-knot, and reniform nematode better than non-mulched fallow in eggplant seedlings. However, amendment of soil with farmyard manure or mahua (*Madhuca indica*) cake before polyethylene mulching did not enhance the solarization (Gaur and Dhingra, 1991). Soil solarization proved superior in terms of weed control (*C. dactylon*) and efficiency in soybean–wheat farming system. Other practices like mulching using wheat straw and repeated cultivation with irrigation were closely effective as soil solarization (Das and Yaduraju, 2008). Kumar et al. (1993) reported that polyethylene mulching for about a month reduced plant-parasitic nematodes (*Tylenchus* spp., *Heterodera* spp., *Xiphinema* spp., *Hoplolaimus* spp., *Pratylenchus* spp., and *Rotylenchus* spp.) populations by 90% and, populations of saprophytic nematodes by 60%. But, the nematode returned to similar pre-solarization levels after 70 days.

In summers, soil solarization followed by glyphosate and in rainy season imazethapyr in soybean controlled *C. rotundus* better and increase soybean–wheat yield compared to different control methods in both seasons (Kumar et al., 2012a,b). During 3-year trial, it was found that soil solarization followed by glyphosate (1.0 kg/ha) reduced the dry weight and weed density by 85% in soybean and 65% in wheat (*Triticum* spp.). Soybean seed yield was 62%, and wheat yield was 28% higher compared with other practices such as summer plowing. The soil solarization followed by glyphosate being superior in weed control and productivity proved to be a beneficial practice in soybean–wheat system, but due to its high cost it was not as economical as the smother crop (cowpea) (Kumar and Das, 2008). Solarization mulching for 1 month decreased the seed emergence of weeds *Dactyloctenium aegyptium, A. racemosa, C. rotundus*, and *Trianthema monogyna* by over 90% in soybean. Sixteen-day of mulching also decreased the wild plants emergence, but it was less effective than 32-day mulching treatment. Plasticulture also increase the soybean growth, while seed yield was also affected (78% of increase) following soil solarization (Kumar et al., 1993).

Plasticulture with polyethylene film significantly increased NO_3_^-^ and NH_4_^+^ nutrients in comparison with untreated soils. Thirty days of soil solarization strongly control seeds of many wild plants such as *Phalaris minor* and *Avena fatua*, whereas the treatment was poorly effective against *Asphodelus tenuifolius* and *T. monogyna*. Such different effect on weeds can be esteemed calculating the ratio of seed germination in solarized condition on seed germination in untreated soil. The index, named solarization reduction index, esteemed as easier to control plants with low value (Arora and Yaduraju, 1998).

#### Tamil Nadu

Tamil Nadu is sited in the southernmost part of Indian Peninsula. Paddy and pulses are two main crops grown in this state, but big production of turmeric, banana, mango, coconut, groundnut, and natural rubber are also recorded. It is also the third largest sapota, coffee, tea, and sugarcane producer. The production of sugarcane is unbeaten in Tamil Nadu. In the central part of the state, average temperatures range from 23 to 37°C during the months of May to June. The average temperature during the months of May to June range from 23 to 37°C. There is lot of scope of soil solarization in this state based on temperatures and crop type.

UV-stabilized transparent polyethylene sheets of 200 μm thickness were laid on clay-loam soil (late spring) at Chidambaram Taluk. At soil depths of 10 and 20 cm, the temperature raised by 65 and 50% respectively, compared to untreated soils. Soil solarization integration with other management option was found effective for pest control (Gill and McSorley, 2010). Significant reduction (up to 55%) in rhizosphere population of *Pythium* spp. causing tomato damping-off was observed in treated soils and which was further reduced to >82% by application of biocontrol agents (BCAs), *T. harzianum* and *P. fluorescens*. Disease incidence was also reduced by >83% in combination with soil solarization and BCAs (Jayaraj and Radhakrishnan, 2008).

Plastic polyethylene mulches were found effective in minimizing weed infestations and increasing yield of groundnut pods. However, the net benefit: cost ratio did not differ between treatments (soil solarization and pre-planting addition of herbicides in flat-bed system) (Subrahmaniyan et al., 2002).

Hyderabad is located in southern part of Telangana state in peninsular India. Hyderabad has a tropical wet and dry climate. Most of the annual rainfall is received in June to September months. Maximum and minimum air temperature of 39 and 24°C are reported for the months of May to June. Cotton, paddy, maize, and groundnut are the major plants grown in this area and vertisol is the common soil type in this state. Solarization studies were conducted mainly on pigeon pea and chick pea at ICRISAT located in this city in 1990s, still there is lot of scope to work on other corps in this state.

Soil was covered with transparent polyethylene sheets of 100 μm thickness for 2 months during spring in 2-year trials at Patancheru. Soil temperature increased about 10°C at soil depth of 5 cm. The temperatures higher than 40°C is potentially deadly for most pathogens, and this value was frequently noticed at 5–10 cm depths of solarized soils, while such high-temperature ranges were fewer in non-solarized control treatment (Chauhan et al., 1988). Sharma and Nene (1990) found that plasticulture also reduced the pre-plant nematode population densities. Transparent polyethylene sheets of 100 μm thickness, for 1.5 month during the summer months reduced the nematodes (*Helicotylenchus retusus, Heterodera cajani, Pratylenchus* sp., *R. reniformis*, and *Tylenchorhynchus* sp.) populations which are pathogens for chickpea and pigeon pea crops. Decrease in population density (93%) was observed for eggs and juveniles of *H. cajani*, while higher values were recorded for *Pratylenchus* spp., *H. retusus*, and *R. reniformis* (98, 99, and 100%, respectively) by 6–10°C increase in soil temperatures in 0- to 20-cm soil depth. Irrigation of soil before covering with polyethylene sheets improved the soil solarization effectiveness and reduced the *H. cajani* population. Soil solarization greatly increased seed yield and dry matter of pigeon pea. The residual effects of solarization on seed yield and total dry matter were not significant.

A further study was conducted at Patancheru, for a week in June. In tropics and sub-tropics, bruchids (*Callosobruchus* spp.) are main post-harvest threat of legumes plants including pigeon pea cultivar and cause significant economic losses. During the period of experimentation, solar irradiance elevated the temperature to 65°C in seed bags. No damage on bruchids was observed in seeds treated with plasticulture after 41 weeks of post-harvest conservation, and no detrimental effect was observed on germination. Conversely, damage of bruchids involved up to 91% of seeds, and reduction of germination (42% of decrease) was observed. Growers in these regions should consider plasticulture as an environmentally safe and cost-saving method to protect pigeon pea seeds (Chauhan and Ghaffar, 2002). Chauhan et al. (1988) reported that *Fusarium* wilt disease was effectively controlled in the susceptible genotypes of pigeon pea and chickpea. However, N-fixation and nodulation were adversely affected due to decline in population of *Rhizobium* with solarization. Due to compensatory effect of increased soil nitrate, plant development and production per hectare were not adversely affected. Even in wilt-resistant genotypes of both pigeon pea and chickpea crops, mainly of pigeon pea, there was a significant increase in yield other than disease control.

#### Gujarat

Gujarat, a home-state of Mahatma Gandhi, is on the western coast of India. Main cultivated plants of this state are groundnut, dates, cotton, and sugarcane. Cereals, pulses, oilseeds, and cotton are widely cultivated; fruit trees and vegetables also have significant percent in cultivated crops. It is also a major producer of milk and milk products. International Food Policy Research Institute reported the annual growth rate of 28% between 2000 and 2008. Winters are dry, mild with an average daytime temperature of 29°C and a nighttime temperature of 12°C. Summers are scorching and dry with a day high and low above 38 and 30°C, respectively.

Patel et al. (1991) found that tarping the beds with clear LDPE film (400 gauge) for 2 months during hot summer months reduced root-knot disease by 58% in Gujarat. Patel et al. (1995) found tarping the beds with clear LDPE plastic of 100 gauge (25 μm) for 15 days during summer to be as effective as 400 gauge (100 μm) for 60 days in getting higher number of bidi tobacco transplants and incremental cost–benefit ratio.

#### Karnataka

This state is in south western region of India. Climate is subtropical, with summer temperatures reaching 40°C in the interiors of the state. Most solarization studies have been conducted on groundnut, French beans, and tomatoes; still there is lot of scope of solarization in other crops.

A combination of solarization and hand weeding was effective for decreasing weeds and increasing groundnut pod yield compared to other treatments which includes solarization along, hand weeding and Alachlor chemical (Soumya et al., 2004). The maximum weed reduction was obtained by transparent plastic film of 0.05 mm thickness for 60 days in wet soils, and maximum yield (28.75 q/ha) was also recorded in groundnut (Biradar and Hosamani, 1997).

The extent of rise in temperature ranged from 8 to 12°C at 5 cm and 7.2 to 11.6°C at 10 cm of soil depth by transparent polyethylene sheets of 0.5 mm thickness in April. In tomatoes, weed control efficiency was maintained till harvest and it was above 80% and, and the highest yield of 21.6 t/ha was achieved (Kumar et al., 2001). The decrease in weed counts due to integration of glyricide and soil solarization was 77% at 3 months after sowing and 74% at harvest in groundnut, whereas weed reduction was 55% at 55 days after sowing in French bean. Similarly, integrated treatment of soil solarization and crop residues increased the yield and other yield components (Mudalagiriyappa Nanjappa and Ramachandrappa, 2001).

### Arid Zone

#### Rajasthan

With biggest desert of India located in this state, it has a typical arid climate with average day high of 41°C. Uncovered soil temperature frequently reaches deadly ranges (up to 60°C) (Israel and Lodha, 2004) during summer months. Lot of studies on soil solarization have been conducted in this region, in Jodhpur. Polyethylene mulching of irrigated and amended loamy sand soil raised the temperature to 57 and 50°C at 0–15 and 16–30 cm soil depths, respectively (Lodha et al., 1997).

In a separate trial the soil temperature of unshaded amended pits achieved 41–45°C, however, under shade temperature remained 6.5–11°C lower than corresponding unshaded pits. Maximal air temperatures ranged from 34.5 to 47.2°C during the corresponding period (Lodha et al., 2003). Same research team documented that temperature of amended soil increased by 2.5°C in non-amended soil (42–51°C) at various soil depths. Integration of soil solarization and amendments also increased the soil temperatures compared to plasticulture (1–5°C) or untreated soils (3–13°C) (Israel et al., 2005). Israel et al. (2011) also observed that in mustard-amended polyethylene mulched plots temperatures may exceed 50°C.

So even with the bare soil temperatures reaching at lethal ranges of 50–60°C during summer months, Israel and Lodha (2004) found it to be ineffective for reducing the population of *F. oxysporum* f.sp. *cumini* (Foc) chlamydospores which causes wilt on cumin. One summer irrigation provided the soil moisture which affected the chlamydospores sensitivity to plasticulture (Katan et al., 1976; Lodha, 1995). Various studies demonstrated the weakening of pathogenic propagules by varying degrees of sub-lethal heat treatments (Freeman and Katan, 1988; Mawar and Lodha, 2009). Lodha and Solanki (1992) found that polyethylene sheets of 50 μm thickness reduced sclerotia inoculum of *M. phaseolina* by 98% of sclerotia per gram of soil at 5 cm soil depth, but effects were milder at deeper soil layers. It also resulted in 30% increase in seed yield of Guar crop.

Combining soil solarization with urea (20 kg nitrogen/hectare) and farmyard manure (10 t/ha) control *F. oxysporum* f.sp. *cumini*, reducing propagules by more than 92% at topsoil layer. The effects on propagules decreased with increasing soil depth, but the reduction was still 74% in wet mulched and amended soil and 56% in dry mulched soil at 30 cm soil depth compared to non-solarized plots. Similarly, the sclerotial population of *M. phaseolina* was reduced by 85% in wet nitrogen manure solarized plots compared to 69% in dry solarized and 10% in non-solarized plots. Seed yield of the following cumin and guar crops were also increased significantly (Lodha, 1995).

Ninety days of dry soil sub-lethal heating was not effective in reducing viable propagules of *M. phaseolina*. But even one summer irrigation with and without sub-lethal heat can reduce the viable pathogen propagules by 43 and 34%, respectively. They also found that amendment of irrigated soil with *Brassica* spp. give 60–72% reduction in viable pathogen propagules, but its effect gets further enhanced to 89–96% on combining with sub-lethal heating. Plastic mulching alone was on par with one summer irrigation of oil-cake amendment and mustard residues (Mawar and Lodha, 2002; Lodha et al., 2003; Israel et al., 2005). Similar results involving the use of natural heat only with mustard oil cake and irrigation have also been reported by Lodha et al. (1997).

Integration of residues of Indian mustard with one irrigation and without plasticulture caused a 70–80% of reduction of the population of *F. oxysporum* f.sp. *cumini* due to the effect of hot summers on soil temperature of amended irrigated, reaching more than 40°C at topsoil layer (Mawar and Lodha, 2002). Interesting results were obtained in terms of residual effect of soil solarization, because a high rate of control of wilt (60% of reduction) was observed in the following winter. The effectiveness of amendments was improved when soils were previously treated with plasticulture, due to thermal stress caused by the treatment to the pathogens (Lodha et al., 2003; Israel et al., 2005; Mawar and Lodha, 2009).

Reduction of incidence of *M. phaseolina* and *F. oxysporum* f.sp. *cumini* pathogen was achieved at topsoil layer as compared to deeper ones (Lodha et al., 1997; Israel et al., 2005). Based on these results, this group suggests the *Brassica* spp. residues are a user-friendly alternative choice to plastic mulches to control soil-borne pathogens in hot, arid regions. From another set of experiments, Israel et al. (2011) suggests that the use of residues produced by farming practices (i.e., obtained from *Verbesina* plants), integrated by irrigation during hot periods and in combination with prolonged heating in managing *F. oxysporum* f.sp. *cumini*. Bohra et al. (1996) observed as root rot incidence of jojoba seedlings caused by *F. solani* was 3% in solarized plots compared to 13% in non-solarized plots. They also reported complete eradication of viable propagules of fungi, *Cylindrocarpon lichenicola* at 20 cm soil depth in solarized plots and no root rot and more vigorous growth of jojoba seedlings till 4 months.

## Widespread of Soil Solarization in India: From Trials to Applications

### Conventional vs. Novel Protection Methods: The Role of Political Commitment to Widespread Soil Solarization

India is a Federal nation that embraces 29 states plus 7 union territories characterized by very different climatic conditions, food markets, and rural policies. In this review, we report findings of solarization trials carried out in 15 states, thus large parts of national territory was untested for potential application of this technique. Conventional treatment methods such as chemicals are easier to use and their effectiveness is less dependent to environmental factors than biological or agronomical methods. Thus introduction or widespread of novel chemicals among farmers generally rely in solid trust in this approach, particularly in areas in which multifunctional role of agriculture is not so widespread. In 2013, only 0.6% of farming territories are certified as organic production, although common polity states strongly encourage the use of low chemical input (Agarwal, 2014). Conversely, solarization successful strongly rely in proper technical executions and other factors difficult to control such as weather conditions, water disposal, and soil properties. Thus, just suggest uncommon pest control methods may lead to failed policy due to (a) lack of efficiency of method due to peculiar environmental conditions and (b) farmer’s distrust in a mostly unknown practices. This latter option should be more common in underdeveloped productive areas of India, where farmers’ propensity toward risk is limited. In fact, some consideration about potential widespread of soil solarization in India may be related to economic factors. Economic issues are not only related to final consumers, but also to investors and farmers. Growers’ propension toward risk exposure was strike by food safety emergency and recession in late 2000s, increasing costs of agricultural products and foods (Maetz et al., 2011), causing negative effects on worldwide agriculture and, in particular, to Asiatic farming (Shane et al., 2009; FAO, 2010). In this contest, the use of low-investment crop protection technique such as solarization seems appropriated, thanks to limited need of mechanization and additional input than plastic films and water.

To popularize adoption of soil solarization in horticulture, the Indian government constituted the National Committee on Plasticulture Applications in Horticulture (NCPAH) and 22 Precision Farming Development Centres has been established in India to promote solarization by undertaking trials, workshops, and training programs in different agriculture climate zones of the country. Besides the predominant application of conventional protection methods, this kind of political commitment seems necessary to develop a systematic knowledge transfer to local producers, increasing confidence among farmers toward soil solarization.

### Perspective on Impact of Solarization on Food Production and Environment

Beside exact method of production the changes in agriculture in the next years can cause significant effect on food security. Analysis of climate risks for crops indicates that South Asia is one of the regions most exposed to the adverse impact on many plants that are relevant to inhabitants exposed to food safety risks (Lobell et al., 2008). In South Asia, estimations of production impacts from climate change in 2030 indicate severe effects on crops that represent the baseline of the human diet in the area, such as rice, wheat, maize, groundnut, or millet (Lobell et al., 2008). Predictions of food demands in 2050 indicate the need of drastically increase the food-related yields worldwide, which results in a more than 2% of increase of rate of production per year (Pingali, 2006). Conversely, the production rates of the most widespread plants in the world (wheat, rice, maize, soybean) are positioned in the range of 0.9–1.6% of increase per year, thus the gap from the predicted needs in the next decades is not so easy to fill (Ray et al., 2013). In India the production of rice is increasing of just 1.0% per year, while increase in Indian wheat yield is set at 1.1% per year. These rates may be not sufficient for Indian development, leaving unaltered the yield per person (Ray et al., 2013). Could soil solarization represent one of the tools that can contribute to fill the yield gap in India? The environmental conditions of many Indian States indicate that plasticulture is an efficient techniques to protect plants and support production. This evaluation is also supported by experimental trials carried out in various States, suggesting how soil solarization could be applied beyond actual contests. Higher levels of soluble minerals nutrients and nitrate-nitrogen were found in solarized soil. Parasitic weed, broomrape (*Orobanche* spp.), and rainy and winter season annuals are adequately controlled by solarization. In Jabalpur (Madhya Pradesh), the yield increase of about 100–125% in onion, 50–55% in groundnut, 70–75% in *Sesamum*, and 77–78% in soybean have been observed using soil solarization (Success Story, Yadav, ICAR, New Delhi, India). These increments could drastically change food availability in India and whole South Asia in the next years if solarization will be widespread.

Environmental impact can assume a significant role in widespread of solarization in India, due to the rapid increase in agriculture-related pollution in the Indian subcontinent. Global interest in physical soil treatment methods is rising due to the progressive elimination of methyl bromide as main method to disinfect soils before cultivation (Luvisi et al., 2015) and soil solarization did not impact negatively with air and water pollution, the two major environmental Indian concerns. However, the disposal of plastic film needs to be carefully evaluated if solarization will be more widely applied in India. With regard to the acreage potentially interested by soil solarization in India (according to actual or perspective plant production), the amount of plastic waste could be enormous, while the carbon footprint for film production can also be highly impacting. In this perspective, the use of biodegradable films seems unavoidable, as well as the development of new ones. Further issue for Indian rural development may be linked to the Kyoto protocol, an international agreements which will drive a substantially reduction of fossil fuels usage to appease the negative effects of climate change. Furthermore, to mitigate the negative impacts on economic development, the gross domestic product of India can be increased thanks to bio-based economy (Lee, 2016). The reduction of organic pollution, an increasing threat in India, can be lead by improved production and application of bioplastics that can substitute traditional plastic in many application fields (Philp, 2014). These next-years perspectives require to develop programs of crop protection that can be feasible according to climate change in India and that can be integrated or can support development of bio-industry. While projected temperatures in South Asia for 2030 should help to fit best environmental requirement for soil solarization (temperatures should increase by more than 1°C), a more than 5% of decrease in precipitation (Lobell et al., 2008) should create issue for soil preparation. However, successful of soil solarization trials in arid zone of India such as Rajasthan (Israel and Lodha, 2004; Israel et al., 2005) indicate as low precipitation did not compromise results, while increase of temperatures could support solarization in moist sub-humid zones such as Himachal Pradesh. Furthermore, the Indian perspective in bioplastic production could support application and development of biodegradable films for solarization. Biodegradable films showed a good potential of using in place of plastic films, but further development is needed to obtain performances comparable to conventional techniques (Bonanomi et al., 2008), and plastic waste management of Indian rural area may have benefits from bio-industry development.

Compared to others world areas in which application of biodegradable films cannot be paired with large-scale bioplastic production (i.e., Mediterranean area), India perfectly fits into requirement for soil solarization application and potential bioorganic plastic production, due to large areas in which wheat or maize are cultivated. In a 2050 scenario, the bioplastic will be mainly produced in Asiatic continent, and India is showing the higher rate of increase per year (more than 5%) in the bioplastic sector (Lee, 2016). Thus, a local production and local consumption model should be achievable for biodegradable films, supporting development of national economy.

## Concluding Remarks

Soil solarization trials reported in this review were not systematically carried out within a national framework, whereas some soil solarization benefits (i.e., reduced environmental impact) or economies of scale (i.e., supplying and disposal of plastic films or irrigation systems) are highlighted by its application at large scale. Organizations such as NCPAH are crucial actors for the widespread use of soil solarization among farmers and drive its development in Indian rural policy, with positive contribution in achieving food sovereignty. Food sovereignty model cannot be yet firmly defined by set of policies (Windfuhr and Jonsén, 2005) but it represents an essential topic to current discussion of strategies to achieve food security in rural areas. India is anything that unrelated to this debate and associations of farmers linked to La Via Campesina are present, such as the Karnataka State Farmers association which is the most visible organization in South Asia (Borras, 2008). Opinions against chemical farming and urgency to gain Indian food sovereignty are key-topic of the Vandana Shiva’s Agenda Anna Swaraj (Food Sovereignty) 2020, but low chemical farming is also promoted by Indian rural policy, even effects are limited (Agarwal, 2014).

Soil solarization cannot be considered as a high-tech solution, with interesting perspective in terms of sovereignty. Crop protection using conventional methods tends to bind farmers to agrochemical industry that, mostly, is not hailing from developing countries. Furthermore, the economic recession in late 2000s made technological sovereignty for plant defense hard to achieve, in particular in Countries in which industrialization is not so widespread. Therefore, technological sovereignty for plant protection is not typical for many South Asia countries, while it may represents a useful resource for sustainability. The use of crop protection techniques should be evaluated with regard to novel approach of sustainability in agricultures; among them, agroecology carefully specify the part of technology in food production. As defined by Altieri and Toledo (2011), sovereignty in technology—and in energetic sources—is a crucial part to achieve food independence of agricultural societies. Fundamentals of this theory involve the use of ecosystem richness, considering biodiversity as an effective production factor and strongly limiting—and if possible, excluding—the use of third-parties inputs. The implementation of technological input locally identified can lead to independence in food production and may reduce risk of food safety (Altieri and Toledo, 2011).

Soil solarization may represent crop protection techniques that substantially relay on locally available resources—that can be considered as local as solar radiation—and it does not generate dependence of farmers to agrochemical producers. Furthermore, the contribution of soil solarization to fill the yield gap may play a significant role in achieving Indian food sovereignty. To conclude, the literature reviewed in this paper indicates more than encouraging effects on production and protection of Indian crops, which can be feasible according to climate change in India and that can be integrated or can support development of national bio-industry. Further trials and communication plans can drive farmers’ growth of confidence toward this protection method.

## Author Contributions

HG, IA, and AL conceived the review; HG and IA analyzed the literature data; HG, IA, and AL prepared the manuscript; HG, LDB, and AL edited the manuscript.

## Conflict of Interest Statement

The authors declare that the research was conducted in the absence of any commercial or financial relationships that could be construed as a potential conflict of interest.

## Footnotes

1 http://www.gktoday.in/major-crops-of-india/

2 https://testbook.com/blog/crops-in-india-gk-notes-pdf/
